# Patterns of referrals to regional clinical genetics services for women potentially at above-population level risk of breast cancer

**DOI:** 10.1038/s44276-023-00027-5

**Published:** 2024-01-11

**Authors:** Juliet A. Usher-Smith, Georgia Tooth, Annabel Follows, Abdul R. Badran, Alice Youngs, Andrea Forman, Katie Snape, Antonis C. Antoniou, Marc Tischkowitz

**Affiliations:** 1 Department of Public Health and Primary Care, University of Cambridge, Cambridge, UK; 2 Cambridge University Hospitals NHS Foundation Trust, Cambridge, UK; 3 School of Clinical Medicine, University of Cambridge, Cambridge, UK; 4 Department of Clinical Genetics, South West Thames Centre for Genomics, St George’s University Hospitals NHS Foundation Trust, London, UK; 5 Department of Medical Genetics, National Institute for Health Research Cambridge Biomedical Research Centre University of Cambridge, Cambridge, UK

## Abstract

**Background:**

The National Institute for Health and Care Excellence (NICE) recommends that women in England at above-population risk be offered additional breast screening and, depending on the level of risk, risk-reducing medication or surgery.

**Methods:**

We reviewed the hospital records of GP referrals made to two large genetics services in England between 01/12/2021-30/11/2022 for women aged 18–49 years and suspected to be at above-population level risk for breast cancer. We compared the women referred with the wider population and estimates of the number of women at above-population level risk using published data.

**Results:**

Up to 20% of women referred did not provide sufficient information for a complete risk assessment and over 25% were considered at near-population level risk after assessment. We estimate that only a small fraction (<10%) of those above population level risk are identified and women in areas of lower deprivation are disproportionately represented amongst referrals.

**Conclusions:**

Many women are missing out on potential preventative and risk-reducing interventions for breast cancer and current pathways may be exacerbating existing health inequalities. Better systems for collecting data on family history, improved methods for risk assessment in general practice and more systematic risk assessment of women prior to population-based screening are needed.

## Background

The National Institute for Health and Care Excellence (NICE) recommends that women in England, Wales and Northern Ireland at above-population risk be offered additional breast screening and, depending on the level of risk, risk-reducing medication or surgery [1]. When calculated based on all known risk factors, it is estimated that 17% of women are at moderate (lifetime risk from age 20 ≥ 17 and <30% or 10-year risk between 40 and 50 ≥ 3% and <8%) or high-risk (lifetime risk from age 20 ≥ 30% or 10-year risk between 40 and 50 ≥ 8%) [2]. All those above population level risk are eligible for risk-reducing medication. Women at moderate risk are offered annual mammography from age 40–60 and women at high-risk annual mammography and/or MRI from the age of 20 depending on known mutations and/or probability of being a carrier of a high-risk genetic mutation [1]. Trials and modelling studies have estimated that annual mammography in women above population level risk reduces mortality from breast cancer by 12–29% [3]. Assuming 25% of eligible women offered risk-reducing medication take up the offer, it is also estimated that 11 cases of breast cancer are avoided per 1000 women offered risk-reducing medication [1, 4, 5].

The pathway for identifying unaffected women at moderate- or high-risk of breast cancer begins with women being referred from general practice. Current guidelines recommend that women presenting with breast symptoms or concerns about relatives with breast cancer should have a first- and second-degree family history taken within general practice [1]. Those meeting a set of criteria (Box 1) should then be referred and a third-degree family history and risk assessment conducted within family history or clinical genetics services.

With referral criteria based only on family history, approximately half of women who are at moderate or high-risk based on all known risk factors will not be identified [6]. Additionally, with no formal screening programme to identify women meeting the criteria for referral, identification of women relies on either the women self-presenting with concerns or general practitioners asking about family history. As a combined consequence, in screening age women only 17.5% of those at moderate- or high-risk had been seen in family history or clinical genetics services [6].

Few data are available on referrals from general practice for women suspected to be at moderate- or high-risk of breast cancer under the age at which they become eligible for population-based screening. Previous studies have either included all referrals to genetic clinics or focused on high-risk populations or the appropriateness of referrals [7, 8].

The aim of this study was to investigate referral patterns from general practice to the clinical genetics services in two large regions of England for women who may be at above-population level risk for breast cancer and are under the age of 50 years when they become eligible for population-based screening.

Box 1 Referral criteria from NICE guidelines [1]

- 1 first-degree female relative diagnosed with breast cancer at younger than age 40 years
- 1 first-degree male relative diagnosed with breast cancer at any age
- 1 first-degree relative with bilateral breast cancer where the first primary was diagnosed at younger than age 50 years
- 2 first-degree relatives, or 1 first-degree and 1 second-degree relative, diagnosed with breast cancer at any age
- 1 first-degree or second-degree relative diagnosed with breast cancer at any age and 1 first-degree or second-degree relative diagnosed with ovarian cancer at any age (1 of these should be a first-degree relative)
- 3 first-degree or second-degree relatives diagnosed with breast cancer at any age.
- First- or second-degree relative diagnosed with breast cancer and any of:
  - bilateral breast cancer
  - male breast cancer
  - ovarian cancer
  - Jewish ancestry
  - sarcoma in a relative younger than age 45 years
  - glioma or childhood adrenal cortical carcinomas
  - complicated patterns of multiple cancers at a young age
  - paternal history of breast cancer (2 or more relatives on the father’s side of the family).

- Families in whom a high-risk predisposing gene mutation has been identified (for example, BRCA1, BRCA2 or TP53)

## Methods

### Design

A service evaluation incorporating retrospective case note review.

### Population

We included all new GP referrals between 01/12/2021-30/11/2022 for women aged 18–49 years made to the East Anglian Regional Medical Genetics Service from the Cambridgeshire and Peterborough Integrated Care System (ICS) and to the South West Thames Centre for Genomics from the South West London ICS. Eligible referrals were identified from referral management systems within each service which include details of the source of each referral. We excluded referrals made on the advice of hospital specialists. The Cambridgeshire and Peterborough ICS covers a population of 211,255 women between the ages of 20–49 [9]. The South West London ICS covers a population of 408,070 women between the ages of 20-49 [9], with 169,709 of those within Merton and Wandsworth.

### Care pathway

In both services, the referral is made by letter (i.e, not on a standardised form) from the GP. On receipt, it is triaged by a consultant or senior member of the cancer genetics team. The patient is then asked to provide detailed family history information, by paper questionnaire in East Anglia and via an online app in South West London. Once received, the clinical team may verify key cancer diagnoses in the family history. The patient’s risk of carrying a cancer-predisposing gene is then assessed according to the UK National Genomic Test Directory [10] and the recommended mammographic screening category assessed using the Institute of Cancer Research mammographic screening protocol [11]. If women do not provide the additional family history information, they are discharged from the service, irrespective of the information provided with the initial referral from the GP.

### Data collection

In both regions, we extracted data from the electronic healthcare records on the age, ethnicity and postcode of each woman and the outcome of the referral. For referrals to East Anglia, data for 10% of the referrals were extracted by two study members (GT or AFol). Those referrals were then reviewed alongside a third study member (MT) to identify any discrepancies. The remaining referrals were then reviewed by either GT or AFoll, with any areas of uncertainty reviewed by MT. For referrals to South West London, data for all referrals were extracted by AB with guidance and review from AF and AY. At both sites, women were followed-up until discharge or until 10/07/2023. For referrals to East Anglia, we also extracted data on the pre-assessment risk category from the GP referral letter. These were categorised as ‘Near-population’, ‘Moderate risk’, ‘High risk’ and ‘Unable to classify’ based on NICE criteria [1]. We used deciles of the Index of Multiple Deprivation (IMD) as a marker of socioeconomic status, calculating the decile of IMD for each referral from the postcode [12]. After data extraction was complete, the data were anonymised for analysis.

To allow comparison between the IMD and ethnicity of those women referred and the wider population in each region, we extracted the population (excluding prisoners) aged 16–59 (the closest to the age range of women considered in this study) in mid-2015 living in each IMD decile in each region from published government statistics [13] and the number of females within each ethnic group in 2021 within each region from the Office of National Statistics [14]. For these analyses we included only referrals within South West London ICS from Merton and Wandsworth as GPs from other regions have the option to refer to alternative family history clinics.

### Analysis

The number and outcome of referrals in each region is presented descriptively. We estimated the number of women aged 20–49 expected to enter the population in each region from the number of women between the ages of 20–49 in each region divided by 30. We then estimated the number of expected referrals and those at moderate- or high-risk of breast cancer within a 12 month period from previous estimates that 3.7% of women meet the NICE guidelines for referral [6] and 17% are at moderate- or high-risk based on multifactorial risk [2].

We compared the IMD and ethnicity of those women referred and those identified as at moderate or high-risk with the population within the catchment area of each service using Chi squared tests, with *p* < 0.05 considered statistically significant. To account for the higher incidence of breast cancer in women in areas of lower deprivation, which may result in a higher rate of referrals, we repeated the comparison for IMD after adjusting for the association between breast cancer and deprivation. To do this we used the published incidence rate ratios (IRR) associated with levels of disposable income [15] to inflate the number of women in the population within the catchment area in the top three deciles (least deprived, IRR 1.16) and deflate the number of women in the population in the bottom three deciles (most deprived, IRR 0.92).

We used multivariable logistic regression with age, IMD decile ethnicity (white vs non-white) (East Anglia only), and referral category (East Anglia only) to assess for associations between these factors and being discharged from the service due to not returning the family history form or consent for further validation. Results from those regression analyses are presented as odds ratios (OR) with 95% confidence intervals.

## Results

**Fig. 1:**
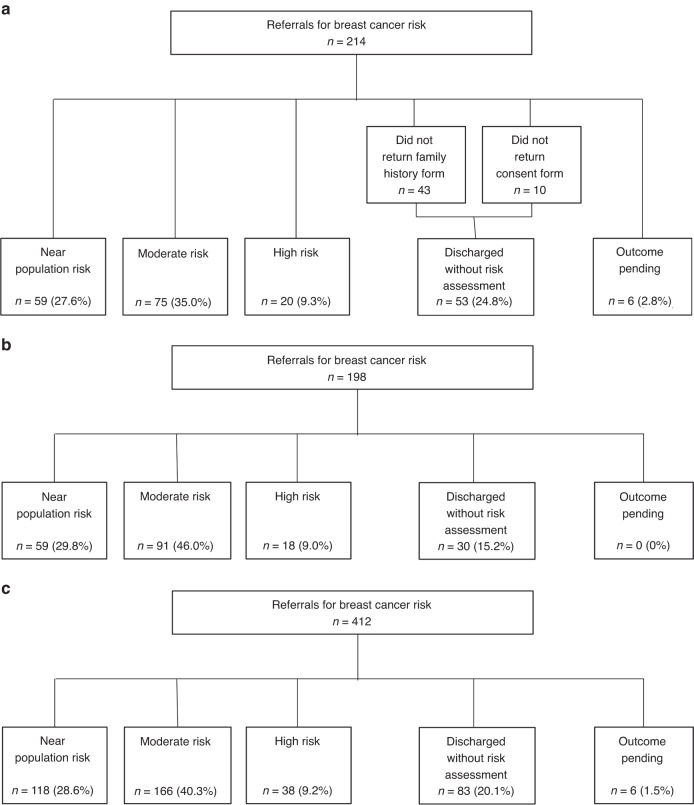
Referrals to genetics services for breast cancer risk and the outcomes of those referrals. **a** East Anglian medical genetics service referrals from Cambridgeshire and Peterborough Integrated Care System; **b** South West Thames Regional Genetics Service referrals from South West London Integrated Care System; **c** Combined referrals to South West Thames Regional Genetics Service and East Anglian regional genetics service from Cambridgeshire and Peterborough and South West London Integrated Care Systems.

Across Cambridgeshire and Peterborough, there were 214 referrals during the study period (Fig. 1a). Of those, 59 (27.6%) were categorised as at near-population level risk, 75 (35.0%) at moderate risk and 20 (9.3%) at high-risk, post-assessment. Fifty-three (24.8%) were discharged back to the care of the GP after either not returning the family history questionnaire or not providing consent to obtain information on other family members. One was diagnosed with breast cancer while awaiting assessment and six (2.8%) were still open at the time of the study.

From South West London there were 198 referrals during the study period (Fig. 1b). Of those, 59 (29.8%) were categorised as at near-population level risk, 91 (46.0%) at moderate risk and 18 (9.1%) at high-risk. Thirty (15.2%) were discharged back to the care of the GP. Ninety-eight of those referrals came from Merton and Wandsworth and so were included in analyses comparing the IMD and ethnicity of the women referred and the wider population. Of these, 65 were categorised as at moderate or high-risk.

There were no associations in either region with being discharged due to not returning the family history questionnaire or family consent and age (OR 1.04 (95% CI 0.98-1.09) in South West Thames and OR 0.76 (0.42–1.34) in East Anglia), or IMD decile (OR 0.86 (0.72–1.03) and OR 1.02 (0.81–1.29)). Additionally, there was no association between being discharged in Cambridgeshire and Peterborough and white vs non-white ethnicity (OR 0.50 (0.06–4.46) or any of the pre-assessment risk-categories extracted from the referral letter (Table 1).

**Table 1:** Characteristics of those completing the risk assessment and those discharged prior to risk assessment.

	Cambridgeshire and Peterborough			South West Thames		
	Completed risk assessment *n* (%)	Discharged prior to risk assessment *n* (%)	Adjusted OR (95% CI)	Completed risk assessment *n* (%)	Discharged prior to risk assessment *n* (%)	Adjusted OR (95% CI)
Age (years)			0.76 (0.42–1.34)			1.04 (0.98–1.09)
18–29	27 (16.8)	8 (15.1)	–	34 (20.2)	5 (16.7)	–
30–39	51 (31.7)	23 (43.4)	–	62 (36.9)	9 (30.0)	–
40–49	83 (51.6)	22 (41.5)	–	72 (42.9)	16 (53.3)	–
Ethnicity						
White	89 (93.7)	33 (97.1)	*Ref*	67 (73.6)	–	–
Non-white	6 (6.3)	1 (2.9)	0.50 (0.06–4.46)	24 (26.4)	–	–
IMD decile			1.02 (0.81–1.29)			0.86 (0.72–1.03)
1	2 (1.3)	5 (9.8)	–	0 (0)	0 (0)	–
2	8 (5.1)	0 (0)	–	7 (4.2)	2 (6.7)	–
3	1 (0.6)	3 (5.9)	–	7 (4.2)	2 (6.7)	–
4	8 (5.1)	2 (3.9)	–	6 (3.6)	4 (13.3)	–
5	15 (9.5)	2 (3.9)	–	16 (9.6)	3 (10.0)	–
6	13 (8.2)	5 (9.8)	–	25 (15.0)	4 (13.3)	–
7	17 (10.8)	8 (15.7)	–	29 (17.4)	3 (10.0)	–
8	28 (17.7)	6 (11.8)	–	27 (16.2)	5 (16.7)	–
9	39 (24.7)	6 (11.8)	–	36 (21.6)	5 (16.7)	–
10	27 (17.1)	14 (27.5)	–	14 (8.4)	2 (6.7)	–
Category of GP referral						
Near-population	42 (26.1)	14 (26.4)	*Ref*	–	–	–
Moderate	59 (36.7)	14 (26.4)	0.90 (0.29–2.82)	–	–	–
High	32 (19.9)	15 (28.3)	1.66 (0.54–5.08)	–	–	–
Unable to tell	28 (17.4)	10 (18.9)	2.64 (0.67–10.34)	–	–	–

*OR* odds ratio for having been discharged, adjusted for all other factors in the table.

*IMD* index of multiple deprivation, where decile 1 is the most deprived and decile 10 the least deprived.

We estimated that 261 and 209 women would meet the NICE criteria for referral and 1197 and 962 would be at moderate- or high-risk in each 12-month period across Cambridgeshire and Peterborough and from the Merton and Wandsworth regions within South West London, respectively. Based on these estimates, 82% (*n* = 214/261) and 47% (*n* = 98/209) of women expected to meet NICE criteria for referral were referred within the two regions and 7.9% (*n* = 95/1,197) and 6.8% (*n* = 65/962) of those at above-population level risk were identified.

Table 2 shows the distribution of ethnicity and IMD decile across the two areas compared with those women referred and those identified as at moderate- or high-risk of breast cancer. In both regions, proportionally more women from areas of lower deprivation were referred and identified at moderate or high-risk (*p* ≤ 0.0001 and *p* = 0.001 for Cambridgeshire and Peterborough and *p* = 0.002 and *p* = 0.008 for Merton and Wandsworth, respectively). This difference persisted after adjustment for the expected increased incidence of breast cancer amongst areas of lower deprivation (*p* = 0.006 for Cambridgeshire and Peterborough and *p* = 0.039 for Merton and Wandsworth). Women of white ethnicity were also over-represented within referrals from both regions but the differences not statistically significant.

**Table 2:** Distribution of ethnicity and socioeconomic status across the two regions compared with those women referred and those identified as at moderate or high-risk of breast cancer.

	East Anglian medical genetics service referrals from Cambridgeshire and Peterborough					South West Thames Regional Genetics Service referrals from Wandsworth and Merton				
	Population *n* (%)	Referrals *n* (%)^a^		Women at moderate/high risk^b^, *n* (%)		Population *n* (%)	Referrals *n* (%)^c^		Women at moderate/high risk^d^, *n* (%)	
Ethnicity			*p* = 0.054		*p* = 0.070			*p* = 0.194		*p* = 0.068
Asian	35,305 (7.80)	3 (2.3)		0 (0)		41,100 (14.5)	7 (7.7)		2 (3.3)	
Black	9380 (2.07)	1 (0.8)		0 (0)		30,790 (10.9)	6 (6.6)		4 (6.6)	
Mixed	13,385 (2.96)	1 (0.8)		1 (1.6)		17,255 (6.1)	6 (6.6)		5 (8.2)	
White	387,260 (85.59)	122 (94.6)		61 (98.4)		181,460 (64.2)	67 (73.6)		46 (75.4)	
Other	7,105 (1.57)	2 (1.6)		0 (0)		12,165 (4.3)	5 (5.5)		4 (6.6)	
IMD decile			*p* < 0.0001		*p* = 0.001			*p* = 0.002		*p* = 0.008
1	19,229 (4.0)	7 (3.4)		1 (1.1)		0 (0)	0 (0)		0 (0)	
2	43,676 (9.0)	8 (3.8)		4 (4.4)		12,435 (3.52)	9 (4.6)		5 (4.6)	
3	26,725 (5.5)	4 (1.9)		0 (0)		31,433 (8.89)	9 (4.6)		5 (4.6)	
4	32,621 (6.7)	10 (4.8)		4 (4.4)		40,774 (11.5)	10 (5.1)		4 (3.7)	
5	54,392 (11.2)	17 (8.1)		10 (10.9)		35,183 (10.0)	19 (9.6)		8 (7.4)	
6	61,017 (12.6)	18 (8.6)		7 (7.6)		58,586 (16.6)	29 (14.7)		18 (16.7)	
7	53,160 (11.09)	25 (12.0)		10 (10.8)		54,740 (15.4)	32 (16.2)		17 (15.7)	
8	65,035 (13.4)	34 (16.3)		12 (13.0)		46,438 (13.1)	32 (16.2)		22 (20.4)	
9	67,750 (14.0)	45 (21.5)		26 (28.3)		44,801 (12.7)	41 (20.8)		23 (21.3)	
10	60,327 (12.5)	41 (19.6)		18 (19.6)		28,994 (8.2)	16 (8.1)		6 (5.6)	

*IMD* index of multiple deprivation, where decile 1 is the most deprived and decile 10 the least deprived.

^a^85 missing data on ethnicity and 5 for IMD.

^b^33 missing data on ethnicity and 3 for IMD.

^c^107 missing data on ethnicity and 1 for IMD.

^d^48 missing data on ethnicity and 1 for IMD.

## Discussion

In this study of referrals for women suspected to be at moderate- or high-risk of breast cancer across two regions of England we show that up to one fifth of women referred did not provide sufficient information for a risk assessment to be completed and over a quarter (36.5% of those who had a complete risk assessment) were considered at near-population level risk. We further estimate that only a small fraction (<10%) of those thought to be at moderate- or high-risk of breast cancer are identified via current routine care and, even after accounting for the increased risk of breast cancer amongst women in areas of lower deprivation, more women in areas of lower deprivation are referred than in areas of higher deprivation.

The small fraction of those estimated to be at moderate- or high-risk identified through current routine care suggests that substantial numbers of women are missing out on potential preventative and risk-reducing interventions. In the FH01 study, it was estimated that 7.8 breast cancer deaths are prevented in women at moderate risk aged 40–49 years per 10,000 screening episodes. [16] Based on the number of women aged 40–49 within Cambridgeshire and Peterborough ICS and South West London ICS alone, we conservatively estimate that up to 232 breast cancer deaths would be prevented in this population if the all women at moderate- or high-risk were identified and took up the offer of annual mammography. It is possible that some of these women had been referred but were discharged before having a complete risk assessment. However, even if all the women discharged without a full risk assessment were at moderate- or high-risk, only 12.4% (*n* = 148/1,197) and 8.1% (*n* = 78/962) of those estimated to be at moderate- or high-risk in the Cambridgeshire and Peterborough ICS and in Merton and Wandsworth, respectively, would have been identified. It is also possible that some women presented to their GP and were not subsequently referred. We suggest that this is also a very small group and that the main reasons women are not identified as at moderate- or high-risk are because the current referral criteria incorporate family history alone and current practice relies on women self-presenting with concerns. Identifying a greater proportion is therefore likely to require a more proactive and systematic approach [17].

Our finding that women from areas of lower deprivation were over-represented in referrals also suggests that the reliance on women self-presenting with concerns is contributing to health inequalities. Such a negative association between referral and deprivation has also been observed at practice level for multiple conditions [18]. The reasons for this are likely to be multifactorial and include individual, provider, system, and policy level factors that contribute to make accessing care for those at higher deprivation more difficult [19]. A proactive and systematic approach alone is unlikely to completely mitigate this as uptake of screening and prevention activities is known to be lower amongst those at greater deprivation [20–22];. More active strategies for engagement, including tailored approaches with underrepresented populations, will therefore be needed alongside any systematic pathway.

The high proportion of women discharged prior to completing a risk assessment is comparable with studies across genetic referrals for all diseases in Wales [18] and Australia [23] in which 24.5% and 30.7%, respectively, of patients referred did not return the family history questionnaires. As suggested [18], this may be because the family history questionnaire is long and complicated or due to a lack of required language or comprehension skills. In this study we are able to show that these women were not re-referred within 6-months. There was also no association between being discharged due to not returning the family history questionnaire and the initial referral category in Cambridgeshire and Peterborough. This reinforces that concern that women at high-risk may be missing out on preventative and risk-reducing interventions. Finding ways to improve the process for women is, therefore required.

The high proportion of women estimated to be at near-population level risk following assessment may simply reflect the known poor performance of family history alone in identifying those at moderate- and high-risk. However, it may also reflect challenges in operationalising the referral criteria for GPs. A retrospective audit of GP referrals in Oxford found that 29% (*n* = 12/41) referrals did not meet the criteria for referral and in 24% (*n* = 10/41) there was insufficient information to know if the criteria was met. [24] Improving the efficiency of referrals from primary care is, therefore, likely to require both a more comprehensive risk assessment within general practice and improved education and guidance on referrals. Tools, for example, CanRisk [25], have been developed and could be used within primary care to enable a more comprehensive risk assessment. Primary care professionals are generally positive about the potential benefits, but barriers include the time needed to complete the assessment, the tool’s compatibility and integration with existing primary care IT systems, competing priorities, and the need for training and capacity building [26]. These barriers will need to be addressed before more comprehensive risk assessment can be implemented. The most appropriate individuals to conduct the risk assessments is also not known and nurses or other members of the healthcare team may be better placed to take on the role than GPs [27, 18, 19]

Strengths of this study are the inclusion of two large regions of England, together covering a population of 619,325 women aged between 20–49 years, and the use of individual level and regional data on ethnicity and deprivation to compare the characteristics of women referred with the population in the two regions. A key limitation is that we do not have data on those women who present to general practice but are not referred. Addressing barriers to referral from general practitioners, including training needs and lack of awareness of genetic services, will also be important [28].

Overall, our findings demonstrate that current practice results in substantial numbers of women missing out on potential preventative and risk-reducing interventions for breast cancer and may be exacerbating existing health inequalities. There is a need for better systems for collecting data on family history, improved methods for risk assessment in general practice and more systematic risk assessment of women under the starting age of population-based screening.

## Data Availability

Anonymised data will be available via the University of Cambridge Data Repository (https://www.repository.cam.ac.uk). Formal requests for access will be considered via a data‐sharing agreement that indicates the criteria for data access and conditions for research use and will incorporate privacy and confidentiality standards to ensure data security.
